# Incidence and factors associated with active tuberculosis among people living with HIV after long-term antiretroviral therapy in Thailand: a competing risk model

**DOI:** 10.1186/s12879-022-07332-3

**Published:** 2022-04-07

**Authors:** Sivaporn Gatechompol, Jiratchaya Sophonphan, Sasiwimol Ubolyam, Anchalee Avihingsanon, Frank van Leth, Frank Cobelens, Stephen J. Kerr

**Affiliations:** 1 HIV-NAT, Thai Red Cross AIDS Research Centre (TRCARC), 104 Ratchadamri Rd., Pathumwan, Bangkok, 10330 Thailand; 2 Center of Excellence in Tuberculosis, Faculty of Medicine, Chulalongkorn University, Bangkok, Thailand; 3 Faculty of Science, Department of Health Sciences, Vrije Universiteit, Amsterdam Public Health Research Institute, Amsterdam, The Netherlands; 4 Department of Global Health, Academic Medical Center, Amsterdam Institute for Global Health and Development, University of Amsterdam, Amsterdam, The Netherlands; 5 The Kirby Institute, University of New South Wales, Sydney, NSW Australia; 6 Biostatistics Excellence Centre, Faculty of Medicine, Chulalongkorn University, Bangkok, Thailand

**Keywords:** TB incidence, Long term ART, PWPH, Competing risk model

## Abstract

**Background:**

Antiretroviral therapy (ART) is known to reduce tuberculosis (TB) incidence among people living with HIV (PLWH). However, studies describing the impact of long-term ART and CD4 count recovery on TB incidence remain scarce due to limited follow up in previous studies. We evaluated TB incidence in a long-term cohort of PLWH on ART in Thailand.

**Methods:**

We conducted an analysis of PLWH aged ≥ 18 years who started ART between 1996 and December 2020. Participants were followed up every 6 months for routine HIV care. TB risk factors, body mass index (BMI), physical examination and full differential blood counts were evaluated at each clinic visit, and CD4 cell counts and HIV RNA every 12 months. Participants diagnosed with TB > 3 months after starting ART were classified as incident cases. Time to event models with death as a competing risk, were used to derive the TB cumulative incidence function (CIF) after ART initiation, and assess time-updated factors associated with incident TB using a six month lag.

**Results:**

A total of 2,636 PLWH contributing 24,229 person years (PY) of follow-up on ART were analysed. Median age was 32.0 (IQR 27.4–37.6) years; 67.5% were male. Median CD4 cell count at ART initiation was 264 (IQR 167–379) cells/mm^3^ and median follow-up duration was 7.6 (IQR 1.9–15.7) years. During follow-up, 113 PLWH developed TB. The probability of incident TB was 0.7%, 1.7%, 3.3% and 4.3%, at 1, 2, 5 and 7 years after ART initiation, respectively. TB CIF was highest among participants with CD4 < 50 cells/mm^3^. The overall crude incidence of TB was 4.66 (95% CI 3.87–5.60) per 1000 PY. Low CD4 count, BMI < 18 kg/m^2^, and substance use in the previous six months were significantly associated with incident TB. Incidence declined with time on suppressive ART, but remained higher than the Thai general population 7 years after ART initiation (2.2 vs 1.5/1000 PY, respectively).

**Conclusion:**

Despite a marked reduction in TB incidence following ART, ongoing TB risk remains high among PLWH, despite long-term suppressive ART. Those with low CD4 cell counts, who are underweight, or currently having substance abuse should be carefully monitored.

**Supplementary Information:**

The online version contains supplementary material available at 10.1186/s12879-022-07332-3.

## Background

Antiretroviral therapy (ART) use has led to a marked reduction in mortality among people living with HIV (PLWH) worldwide [1, 2]. However, tuberculosis (TB) remains a common opportunistic infection and a major cause of death among PLWH. In 2019, an estimated 1.4 million people died from TB worldwide, including 208,000 PLWH [3]. In high TB burden countries, ART is associated with a significant reduction in TB incidence [4, 5]. A systematic review and meta-analysis showed that ART reduced the incidence of TB by 65% across all levels of CD4 count [6]. PLWH initiating ART are at higher risk of experiencing TB during the first 3 months of treatment; the risk declines thereafter [6–9]. However, most relevant studies have a short follow-up; with very few of > 5 years. Moreover, most previous studies were done in sub-Saharan Africa, and may not be applicable to Asia where the TB and HIV burden are different.

In 2019, an estimated 9.5% of the 105,000 incident TB cases in Thailand were co-infected with HIV, and 16.5% of the 11,500 deaths among PLWH were due to TB [3]. Although the universal coverage system has provided free HIV testing and ART for PLWH regardless of CD4 cell count since 2017, only 82% of TB/HIV co-infected people were on ART in 2019 [10]. Despite the high burden of TB in Thailand, little information is available regarding the epidemiology of TB among PLWH on long-term ART.

In this study, we aimed to investigate the incidence of active TB, and evaluate the factors associated with incident TB among participants in the HIV-NAT observational cohort (HIV-NAT 006 cohort), the longest, ongoing HIV observational cohort in Thailand.

## Methods

### Study cohort and population

This is a post-hoc analysis of the data from the HIV-NAT 006 cohort. This prospective, clinic-based cohort has enrolled adults living with HIV aged ≥ 18 years since 1996 (Clinicaltrials.gov NCT00411983). The participants are seen every 6 months at HIV-NAT, Thai Red Cross AIDS Research Centre, Bangkok. Follow-up care for HIV infection and ART are provided according to relevant Thai treatment guidelines [11–14].

Prior to 2010, ART was initiated in PLWH with CD4 cell counts < 200 cells/mm^3^ regardless of symptoms. In 2010, the CD4 threshold criteria for initiating ART was changed to < 350 cells/mm^3^. In 2014, the CD4 criteria to start ART was changed to < 500 cells/mm^3^. Later, in 2017, ART was initiated at any CD4 count. The standard first-line ART included 2 nucleoside reverse transcriptase inhibitors (NRTs; zidovudine, stavudine, lamivudine or tenofovir) plus 1 non-nucleoside reverse transcriptase inhibitor (NNRTIs; nevirapine or efavirenz). The following variables were collected at each clinic visit: body temperature, body weight, body mass index (BMI), physical examination findings, full differential blood counts, lipid profile, creatinine, and alanine aminotransferase (ALT). CD4+ and CD8+ lymphocyte counts as well as HIV RNA were done every 12 months.

Most HIV-NAT 006 cohort participants had previously participated in clinical trials, and after completion of the trials, they were automatically enrolled into the cohort to receive continuous care. For the present analysis, we included cohort participants aged ≥ 18 years who initiated ART from 1996 to December 2020. Participants who initiated ART before enrolling into HIV-NAT 006 were included in the analysis if all relevant clinical information was available at the time of enrolment. Follow-up data up to June 2021 were analyzed so all of the participants had ≥ 6 months of follow-up.

### Ethical approval

This cohort was approved by the medical ethics committee, Faculty of Medicine, Chulalongkorn University, Bangkok. This study was conducted according to the Declaration of Helsinki and Good Clinical Practice. All participants provided written informed consent.

### Definitions and outcomes

The primary outcome was TB incidence after ART initiation. All participants were screened for TB prior to starting ART. Participants diagnosed with active TB 12 months before ART initiation, or within 3 months of starting ART were suspected to have unmasking of TB-associated immune reconstitution inflammatory syndrome (TB-IRIS) and were excluded from the analysis. During follow-up, participants with symptoms and/or signs of pulmonary or extrapulmonary TB were assessed as per relevant National TB treatment guidelines [15, 16] which included physical examination, chest radiography, sputum smear microscopy, culture for *M. tuberculosis* and/or GeneXpert MTB/RIF (Xpert; Cepheid, Sunnyvale, CA) as indicated.

Active TB was defined as a participant who presented with symptoms and signs suggestive of TB and received a course of TB treatment.

A diagnosis of incident TB case was defined as (1) active TB bacteriologically confirmed by smear microscopy, culture or Xpert, or (2) active TB clinically diagnosed based on radiologic evidence of TB without bacteriological confirmation and with good clinical response to antituberculosis treatment. TB preventive therapy was not routinely provided, as recommended by local guidelines.

### Statistical analysis

We used competing risks regression [17] to calculate the subdistribution hazard for developing TB, with death as a competing risk. Risk time began 3 months after ART initiation, to align with the exclusion criteria based on unmasking TB occurrence, and ended on the last visit date, date of TB diagnosis, death, or 1 June 2021, whichever came first. Participants lost to follow-up (LTFU) or referred to other hospitals were censored at the last visit date when they were known to be alive and free from TB. LTFU was defined as not attending the clinic for 12 months after their previous appointment. Participants subsequently returning to the clinic before the study period ended were not considered LTFU. The Cumulative Incidence Function (CIF) was used to estimate the probability of TB after ART initiation; subdistribution hazard models were applied to assess covariate effects on the CIF. To facilitate comparison with other studies, we also derived crude TB incidence rates (IR), with numerator of incident TB cases divided by person-time at risk at each time point, and expressed as events per 1,000 person-years (PY) with accompanying 95% confidence intervals (CI).

Time-fixed covariates were age, sex and CDC disease classification at ART initiation. Time-varying covariates were modelled with a 6-month lag, and included viral load (VL), CD4 count, smoking status, alcohol use, substance use, close contact with TB cases, other co-morbidities and BMI. CD4 count was modelled as clinically relevant categories. We used the BMI classification for Asians defined by WHO [18] as follows: underweight as BMI < 18.5 kg/m^2^, normal as BMI 18.5–22.9 kg/m^2^, and overweight or obese as BMI ≥ 23 kg/m^2^. We also conducted two sensitivity analyses: the first was limited to cohort participants with bacteriologically confirmed TB, the second where time up-dated variables were modelled with a-12 month lag, to provide more information on the temporal association of BMI and incident TB.

Covariates significant in univariable analyses with p < 0.10 were adjusted for in multivariable analyses. Associations with p < 0.05 in multivariate models were considered statistically significant. Analyses were done using Stata version 17.0 (StataCorp., College Station, TX, USA).

## Results

### Characteristics of the study participants

**Fig. 1:**
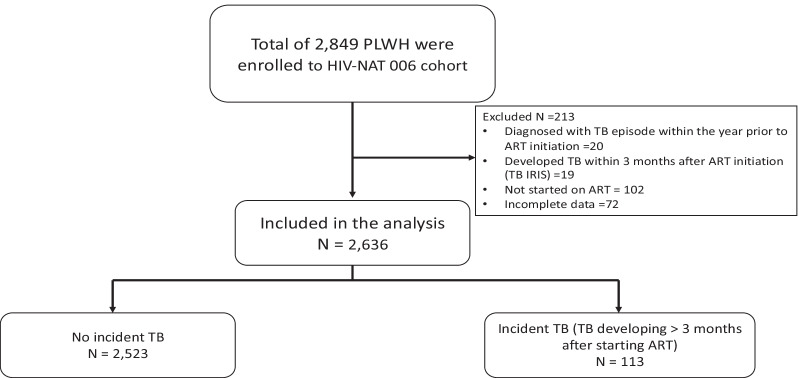
Overview of cases included in the analysis

From 1996 to May 2021, 2,849 PLWH were enrolled into HIV-NAT 006. Twenty participants were diagnosed with TB ≤ 1 year before ART initiation, 19 developed TB within 3 months (TB IRIS), 102 participants did not start ART and 72 had incomplete baseline data so were excluded. Hence, 2,636 PLWH were included in the analysis (Fig. 1) and contributed 24,229 person years of follow-up on ART. There are 130 participants died during the follow up period.

The median age at ART initiation (baseline) was 32.0 (IQR 27.4–37.6) years, and 1,791 (67.6%) were male. Median baseline CD4 cell count was 264 (IQR 167–379) cells/mm^3^ and median follow-up duration was 7.6 (IQR 1.9–15.7) years. At baseline, 203 (7.7%) participants had CD4 counts < 50 cells/mm^3^; hepatitis B surface antigen (HBsAg) and anti HCV antibody were positive in 13.9% and 9.5% of the participants, respectively. Participants characteristics at ART initiation are shown in Table 1.

**Table 1:** The participants’ characteristics at ART initiation

Characteristic	Total (N = 2,636)
Age (years), median (IQR)	32.0 (27.4–37.6)
Males, N (%)	1791 (67.5)
BMI (kg/m^2^), median (IQR)	21.7 (19.7- 24)
CDC classification C, N (%)	326 (12.3)
CD4 cell count (cells/mm^3^), median (IQR)	264 (167–379)
HIV-RNA (log_10,_ copies/ml), median (IQR)	4.5 (3.9–5)
Current smokers, N (%)	354 (20.2)
Currently using alcohol, N (%)	291 (14.6)
History of substance use, N (%)	111 (6.6)
Diabetes Mellitus, N (%)	140 (5.3)
Hypertension, N (%)	364 (13.7)
Hepatitis B co-infection, N (%)	368 (13.9)
Hepatitis C co-infection, N (%)	253 (9.5)

*ART* antiretroviral therapy, *BMI* body mass index, *N* number, *IQR* interquartile range

### Incident TB during follow-up

During the follow-up period, 113 PLWH developed incident TB. Median age at incident TB diagnosis was 37.9 (IQR 32.4–43.3) years, 73 (65%) were male and median CD4 count at ART initiation was 207 (IQR 120–342) cells/mm^3^. At the time of TB diagnosis, 48 (42.5%) participants were virologically suppressed and 65 (57.5%) had virological failure (viral load > 50 copies/ml) while on ART. Six (5.3%) had a history of previous TB. Median time from ART initiation to TB diagnosis was 4.4 (IQR 1.7–7.9) years; pulmonary TB was more common (74.3%) than extrapulmonary TB (25.7%, Table 2).

**Table 2:** Clinical characteristics of participants with incident tuberculosis after ART initiation

	Incident TB (N = 113)
Classification of TB, n (%)	
Pulmonary TB	84 (74.3%)
Extrapulmonary TB	
TB lymph node	16 (14.1%)
Disseminated TB	7 (6.2%)
TB pleura	3 (2.6%)
• TB meningitis	1 (0.8%)
TB arthritis/spine	1 (0.8%)
TB abdomen	1 (0.8%)
Diagnosis of TB criteria	
Bacteriologically confirmed	30 (27%)
No resistance	25 (83.3%)
Mono-resistant TB	3 (9.9%)
Multidrug resistant	2 (6.8%)
Clinically diagnosed	52 (47%)
Not evaluated	31 (26%)

*TB* tuberculosis

**Fig. 2:**
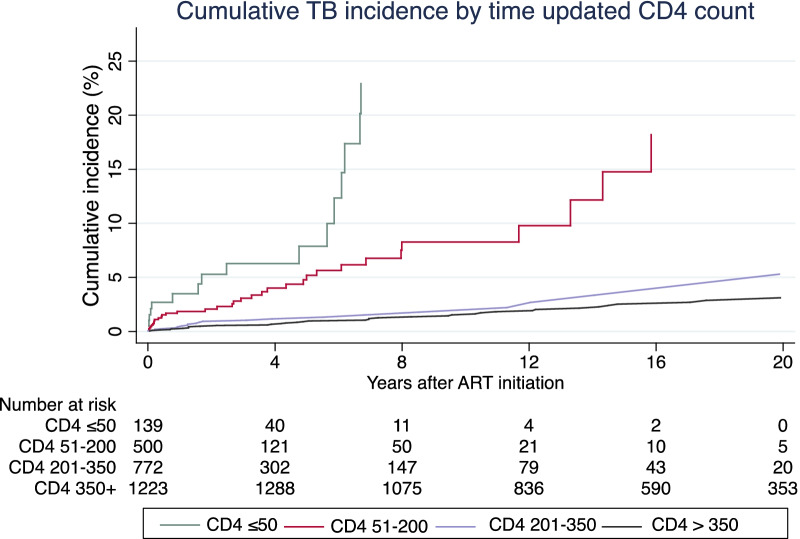
Cumulative incidence of TB after ART initiation stratified by time updated CD4

The cumulative incidence of TB was 0.7%, 1.7%, 3.3% and 4.3%, at 1, 2, 5 and 7 years after ART initiation, respectively. The TB CIF was highest among participants with time updated CD4 < 50 cells/mm^3^ and decreased as the CD4 count increased to 50–200, 201–350 or > 350 cells/mm^3^ (Fig. 2 and Table 3).

**Table 3:** TB cumulative incidence function (CIF) after ART initiation, stratified by time updated CD4 cell count (95% CI)

Time (Years)	Overall CIF (%)	CIF in CD4 < 50 cells/mm^3^ (%)	CIF in CD4 51–200 cells/mm^3^ (%)	CIF in CD4 201–350 cells/mm^3^ (%)	CIF in CD4 > 350 cells/mm^3^ (%)
1	0.7	1.7	0.7	0.4	0.2
2	1.7	3.7	1.2	0.8	0.4
5	3.3	7.5	4.6	1.1	0.8
7	4.3	20.9	6.5	1.6	1.2

The overall crude incidence of TB in our study was 4.66 cases (95% CI 3.87–5.60) per 1000 PY. The crude incidence rates at 1, 2, 5 and 7 years after ART initiation were 10.83 (95% CI 7.81–15.01), 5.99 (95% CI 3.32–10.81), 4.81 (95% CI 2.4–9.63) and 2.16 (95% CI 6.0–9.2) cases per 1000 PY, respectively.

### Factors associated with incident TB after ART initiation

In our multivariable competing risk regression model, factors significantly associated with TB incidence were BMI < 18 kg/m^2^ (Underweight) (aSHR = 8.21, 95% CI 2.43–27.73, p = 0.001) compared to BMI 18–23 kg/m^2^ (Normal weight), history of substance use (aSHR = 6.03, 95% CI 1.16–31.21, p = 0.032), and time updated CD4 count < 50 cells/m^3^ (aSHR = 18.23, 95% CI 3.35–99.16, p = 0.001) or 50–200 cells/m^3^ (aSHR = 5.43, 95% CI 0.98–29.92, p = 0.05) compared to CD4 > 350 cells/m^3^ (Table 4). In our sensitivity analysis including only bacteriologically confirmed incident TB, results were consistent with the primary analysis (Additional file 1: Table S1). In our second sensitivity analysis where we explored the temporal nature of the association of TB with BMI using a 12-month covariate lag, the association with BMI < 18.5 was reduced (aSHR 2.58 (95% CI 1.12–6.03), the association with CD4 count < 50 was increased (aSHR 36.6 (95% CI 9.23–145.03) and the association with substance use was lost (Additional file 1: Table S2).

**Table 4:** Factors associated with incident tuberculosis among participants from a competing risks regression model

	Univariable		Multivariable	
	SHR (95% CI)	P	aSHR (95% CI)	P
At ART initiation				
Males	1.22 (0.86–1.77)	0.300		
CDC classification C	4.02 (2.75–5.88)	< 0.001	1.22 (0.25–5.97)	0.802
History of prior TB	0.78 (0.34–1.78)	0.556		
Time updated variables				
Age	0.98 (0.96–1.00)	0.195		
BMI Classification				
< 18.5 kg/m^2^	9.59 (3.45–26.64)	< 0.001	8.21 (2.43–27.73)	0.001
18.5–23 kg/m^2^	1		1	
> 23 kg/m^2^	0.94 (0.29–3.00)	0.912	0.71 (0.18–2.84)	0.628
Smoking history				
Non-smoker or ex-smoker	1			
Current smoker	1.13 (0.77–1.66)	0.520		
Alcohol consumption				
No alcohol use in past 3 months	1			
Alcohol use in past 3 months	0.84 (0.57–1.24)	0.390		
History of substance use	2.11 (0.91–4.88)	0.078	6.03 (1.16–31.21)	0.032
History of close contact TB cases	1.64 (0.98–2.74)	0.058	1.71 (0.37–7.78)	0.489
Hypertension	1.13 (0.68–1.88)	0.621		
Diabetes mellitus	0.44 (0.14–1.42)	0.171		
Hepatitis B co-infection	0.93 (0.52–1.64)	0.807		
Hepatitis C co-infection	1.39 (0.79–2.42)	0.246		
CD4 cell count (cell/m^3^)				
• < 50	12.02 (5.96–24.22)	< 0.001	18.23 (3.35–99.16)	0.001
• 51–200	8.51 (4.97–14.53)	< 0.001	5.43 (0.98–29.92)	0.052
• 201–350	2.32 (1.31–4.11)	0.004	1.99 (0.35–11.07)	0.432
• > 350	1		1	
HIV-RNA ≥ 50 copies/mL	2.55 (0.53–12.25)	0.243		

*SHR* sub-distribution hazard ratio

## Discussion

In this long-term cohort of PLWH on ART in Thailand the cumulative incidence of TB was 0.7%, 1.7%, 3.3% and 4.3%, at 1, 2, 5 and 7 years after ART initiation, respectively. Using a 6-month lag of potential predictor variables, those significantly associated with developing TB after starting ART were time updated CD4 count, BMI < 18 kg/m^2^, and a history of substance use.

Our study provides estimates of the expected TB incidence in a high TB burden, low to middle-income country, where ART is provided free of charge but TB preventive therapy is not routinely implemented. The overall incidence density was 4.66 cases per 1000 PY. The highest incidence occurred in the first year after ART initiation (10.8 cases per 1000 PY) and decreased to 2.2 cases per 1000 PY after 7 years on ART. This reduction is similar to reports from Africa [9, 19, 20] and India [21]. Nevertheless, despite this reduction, TB incidence after 7 years of ART in our study was higher than the estimated rate among the general Thai population in 2019 (1.5 per 1000 PY) [3]. Our data confirm that TB risk among PLWH remains high, even after long-term ART, and are consistent with those of a previous long-term ART cohort study (median 5.0 years) conducted in Africa [22].

Low CD4 counts were strongly associated with TB incidence, regardless of ART duration. Participants with a recent CD4 count < 50 cell/mm^3^ were at the highest risk for incident TB compared to those with CD4 count above 350 cells/mm^3^, after adjusting for BMI, history of substance use, history of close contact to TB cases and clinical stage at ART initiation. Our findings are similar to those from previous studies in high TB burden settings [22–24]. Even though ART is associated with substantial reductions in TB incidence rates in treated cohorts, it fails to fully restore the immune response against *M. tuberculosis.* This may be due to functional immune defects that persist despite long-term ART [25–27]. Cumulative TB incidence depends on the amount of time that participants spend at low CD4 cell counts during ART. Our data supports rapid ART initiation strategies, which reduce the risk of low CD4 counts.

Our study also found that underweight PLWH (BMI < 18.5 kg/m^2^) had a higher risk of developing TB. This is consistent with HIV cohorts from Tanzania [28], Ethiopia [29] and South Africa [30]. A previous systematic review showed an inverse relationship between BMI and TB incidence: being overweight or obese protected against TB infection [31]. These findings are confirmed by many large non-HIV cohorts in low-medium TB burden countries [32–35]. In our models, BMI with a 6-month lag was a considerably stronger predictor of TB compared to BMI with a 12-month lag. This suggests while low BMI increases the risk of incident TB, weight loss may be a clinical indicator of early, subclinical TB. In support of this, animal studies have shown that adipose tissue serves as an important reservoir for *M.tuberculosis* [36, 37]*.* A recent murine TB model demonstrated that adipose tissue loss enhanced pulmonary TB infection, and increased *M.tuberculosis* load in the lungs of infected mice [38]. In addition to being a risk factor for incident TB [28–30, 39] low BMI also predicts mortality among PLWH [40–42]. In settings with limited diagnostic capacity, BMI may therefore be the simplest predictor to identify individuals who are at high risk of developing TB.

Substance use was also an independent risk factor for TB incidence, consistent with previous studies [43, 44]. TB infection is increased in drug users for a number of reasons including direct impairment of both cell mediated and humoral immune responses [45, 46]. Moreover, the environment and risk behaviors of this population indirectly contribute to higher TB risk due to higher rates of nonadherence to ART and TB treatments, and poor nutrition [47]. For these reasons, a multidisciplinary approach is needed to optimize treatment outcomes in this population.

There are some limitations in our study. First, similar to all retrospective studies, some episodes of TB and other confounding factors may have been unreported, leading to an underestimation of incident TB. Second, our data were collected from a single HIV research clinic in Thailand, which might limit the generalizability of the results. Third, only 27% of incident TB cases had bacteriologically confirmed TB. It is possible that some of the participants who were diagnosed clinically had pathogens other than *M. tuberculosis*. Last, since TB preventive therapy (TPT) was not evaluated in this study we could not demonstrate additional benefits of TPT in conjunction with ART. Nevertheless, limiting the analysis population to these patients gave results that were consistent with the entire cohort. Despite these limitations, to our knowledge, this HIV cohort has the longest follow-up time in Southeast Asia. For more than 25 years, data has systematically been collected and under research conditions. Moreover, we used competing risks models that consider the occurrence of events that preclude TB diagnosis during the follow-up period, thereby providing more accurate incidence estimates compared to other standard models [48].

Although ART has led to a marked reduction in mortality and improved life expectancy of PLWH [4, 5], ongoing TB risk remains high among PLWH, despite long-term ART. To achieve the best clinical outcomes, rapid ART initiation in treatment naïve individuals is important [6], close monitoring for incident TB, especially in underweight patients is necessary, and clinicians should be aware of patients who currently use illicit drugs or have low CD4 cell counts.

## Supplementary Information

**Additional file 1.** **Table S1.** Analyses of subdistribution hazards models for only bacteriologically confirmed cases. **Table S2.** Factors associated with incident tuberculosis among participants from a competing risks regression model (12 months interval).

## Data Availability

The datasets generated and/or analysed during the current study are available from the corresponding author on reasonable request.

## Abbreviations

ALT: Alanine aminotransferase
ART: Antiretroviral therapy
BMI: Body mass index
CIF: Cumulative incidence function
CI: Confidence interval
HBsAg: Hepatitis B surface antigen
IQR: Interquartile range
LTFU: Lost to follow-up
NRTIs: Nucleoside reverse transcriptase inhibitors
NNRTs: Non-nucleoside reverse transcriptase inhibitors
PLWH: People living with HIV
SHR: Sub-distribution hazard ratio
TB: Tuberculosis
TB-IRIS: TB-associated immune reconstitution inflammatory syndrome
TPT: TB preventive therapy
VL: Viral load
