# Supplementary material for: Incidence and factors associated with active tuberculosis among people living with HIV after long-term antiretroviral therapy in Thailand: a competing risk model
Source: BMC Infect Dis. 2022 Apr 7;22:346. doi: 10.1186/s12879-022-07332-3 (PMC8988401; doi:10.1186/s12879-022-07332-3)
Supplement: Supplementary file 1 — Additional file 1. Table S1. Analyses of subdistribution hazards models for only bacteriologically confirmed cases. Table S2. Factors associated with incident tuberculosis among participants from a competing risks regression model (12 months interval). [file 12879_2022_7332_MOESM1_ESM.docx]

**Table S1. Analyses of subdistribution hazards models for only bacteriologically confirmed cases**

|  | **Univariable** | | **Multivariable** | |
| --- | --- | --- | --- | --- |
|  | **SHR (95%CI)** | **P** | **aSHR (95%CI)** | **P** |
| **At ART initiation** |  |  |  |  |
| Male | 1.33 (0.86-1.77) | 0.38 |  |  |
| CDC classification C | 1.48 (0.68-3.24) | 0.32 |  |  |
| **Time updated variables** |  |  |  |  |
| Age | 0.96 (0.92-1.01) | 0.13 |  |  |
| BMI Classification |  |  |  |  |
| - < 18 kg/m^2^ (Underweight) | 3.67 (0.57-23.43) | 0.17 |  |  |
| - 18-23 kg/m^2^ (Normal) | 1 |  |  |  |
| - > 23 kg/m^2^ (Overweight) | 1.15 (0.23-5.78) | 0.86 |  |  |
| Smoking history |  |  |  |  |
| - Non-smoker or ex-smoker | 1 |  |  |  |
| - Current smoker | 0.61 (0.29-1.23) | 0.17 |  |  |
| Alcohol consumption |  |  |  |  |
| - No alcohol use in past 3 months | 1 |  |  |  |
| - Alcohol use in past 3 months | 0.58 (0.12-2.75) | 0.50 |  |  |
| History of substance use | 3.59 (1.03-12.5) | 0.045 | 4.43 (1.17-16.68) | 0.028 |
| History of close contact TB cases | 1.49 (0.89-2.50) | 0.13 |  |  |
| Hypertension | 0.76 (0.23-2.53) | 0.66 |  |  |
| Hepatitis B co-infection | 1.10 (0.49-2.43) | 0.81 |  |  |
| Hepatitis C co-infection | 0.81 (0.30-2.19) | 0.68 |  |  |
| CD4 cell count (cell/m^3^) |  |  |  |  |
| - < 200 | 4.11 (1.84-9.15) | 0.001 | 4.94 (1.70-14.25) | 0.003 |
| - ≥ 200 | 1 |  |  |  |
| HIV-RNA ≥ 50 copies/mL | 4.02 (0.39-40.69) | 0.24 |  |  |

**For the CD4 could not be divided into 4 categories because the sample size was too small

**Table S2. Factors associated with incident tuberculosis among participants from a competing risks regression model. (12 months interval)**

|  | **Univariable** | | **Multivariable** | |
| --- | --- | --- | --- | --- |
|  | **SHR (95%CI)** | **P** | **aSHR (95%CI)** | **P** |
| **At ART initiation** |  |  |  |  |
| Males | 1.26 (0.86-1.77) | 0.24 |  |  |
| CDC classification C | 3.97 (2.72-5.78) | <0.001 | 1.22 (0.25-5.97) | 0.80 |
| History of prior TB | 0.79 (0.35-1.81) | 0.59 |  |  |
| **Time updated variables** |  |  |  |  |
| Age | 0.98 (0.95-1.00) | 0.052 | 0.97 (0.93-1.00) | 0.14 |
| BMI Classification |  |  |  |  |
| - < 18 kg/m^2^ | 3.41(1.83-6.36) | <0.001 | 2.58 (1.12-6.03) | 0.026 |
| - 18-23 kg/m^2^ | 1 |  | 1 |  |
| - > 23 kg/m^2^ | 0.62 (0.33-1.13) | 0.12 | 0.66 (0.33-1.34) | 0.26 |
| Smoking history |  |  |  |  |
| - Non-smoker or ex-smoker | 1 |  |  |  |
| - Current smoker | 1.08 (0.78-1.67) | 0.68 |  |  |
| Alcohol consumption |  |  |  |  |
| - No alcohol use in past 3 months | 1 |  |  |  |
| - Alcohol use in past 3 months | 0.80 (0.53-1.20) | 0.29 |  |  |
| History of substance use | 2.45 (1.05-5.70) | 0.038 | 2.04 (0.62-6.76) | 0.24 |
| History of close contact TB cases | 1.66 (1.00-2.75) | 0.050 | 1.07 (0.40-2.86) | 0.89 |
| Hypertension | 1.03 (0.62-1.71) | 0.89 |  |  |
| Diabetes mellitus | 0.42 (0.13-1.34) | 0.15 |  |  |
| Hepatitis B co-infection | 0.94 (0.53-1.66) | 0.84 |  |  |
| Hepatitis C co-infection | 1.45 (0.83-2.53) | 0.19 |  |  |
| CD4 cell count (cell/m^3^) |  |  |  |  |
| - < 50 | 33.68(16.4-69.03) | <0.001 | 36.60 (9.23-145.03) | 0.001 |
| - 51-200 | 9.55(5.62-16.23) | <0.001 | 4.83 (1.72-13.55) | 0.003 |
| - 201-350 | 2.40(1.37-4.21) | 0.002 | 2.72 (1.32-5.59) | 0.006 |
| - > 350 | 1 |  | 1 |  |
| HIV-RNA ≥ 50 copies/mL | 3.95 (0.88-17.89) | 0.07 | 1.23 (0.12-11.77) | 0.85 |
